# Expression of fluorescent proteins in *Lactobacillus rhamnosus* to study host–microbe and microbe–microbe interactions

**DOI:** 10.1111/1751-7915.12872

**Published:** 2017-10-13

**Authors:** Irina Spacova, Elke Lievens, Tine Verhoeven, Hans Steenackers, Jos Vanderleyden, Sarah Lebeer, Mariya I. Petrova

**Affiliations:** ^1^ Centre of Microbial and Plant Genetics KU Leuven Heverlee Belgium; ^2^ Department of Bioscience Engineering University of Antwerp Antwerp Belgium

**Keywords:** probiotics, fluorescence, mTagBFP2, mCherry, biofilms, adhesion

## Abstract

Probiotic *Lactobacillus* strains are widely used to benefit human and animal health, although the exact mechanisms behind their interactions with the host and the microbiota are largely unknown. Fluorescent tagging of live probiotic cells is an important tool to unravel their modes of action. In this study, the implementation of different heterologously expressed fluorescent proteins for the labelling of the model probiotic strains *Lactobacillus rhamnosus* GG (gastrointestinal) and *Lactobacillus rhamnosus* GR‐1 (vaginal) was explored. Heterologous expression of mTagBFP2 and mCherry resulted in long‐lasting fluorescence of *L. rhamnosus* GG and GR‐1 cells, using the nisin‐controlled expression (NICE) system. These novel fluorescent strains were then used to study *in vitro* aspects of their microbe–microbe and microbe–host interactions. *Lactobacillus rhamnosus* GG and *L. rhamnosus* GR‐1 expressing mTagBFP2 and mCherry could be visualized in mixed‐species biofilms, where they inhibited biofilm formation by *Salmonella* Typhimurium–*gfpmut3* expressing the green fluorescent protein. Likewise, fluorescent *L. rhamnosus* GG and *L. rhamnosus* GR‐1 were implemented for the visualization of their adhesion patterns to intestinal epithelial cell cultures. The fluorescent *L. rhamnosus* strains developed in this study can therefore serve as novel tools for the study of probiotic interactions with their environment.

## Introduction

The ability to specifically label live bacteria is a powerful advantage for the *in vivo* and *in vitro* tracking of their behaviour, survival and interactions with other microorganisms, as well as their potential hosts. Heterologous expression of fluorescent proteins remains one of the most useful and common tools for bacterial imaging (Olenych *et al*., 2007; Dean and Palmer, 2014). This approach results in higher labelling stability and specificity of live single cells compared with externally added dyes, allows prolonged time‐lapse imaging and circumvents the need for exogenous dye internalization.

Currently, a broad selection of fluorescent proteins with varying fluorescence emission spectra and properties is available due to continuous efforts aimed at expanding the spectral range and improving their brightness and photostability (Olenych *et al*., 2007). For example, the green fluorescent protein (GFP) isolated from the *Aequorea victoria* jellyfish was the first fluorescent protein to be discovered and subsequently cloned in various live bacterial cells (Chalfie *et al*., 1994). Improved genetically modified GFP variants in the green (GFPuv) (Crameri *et al*., 1996), cyan (enhanced cyan fluorescent protein, ECFP) (Cubitt *et al*., 1995) and yellow (mVenus) (Nagai *et al*., 2002) spectra have also been developed. At the same time, a new group of fluorescent proteins with other fluorescence emission spectra and efficient maturation at 37°C has been discovered in reef corals and sea anemones, including the monomeric red spectrum protein mCherry (Shaner *et al*., 2004) and the blue spectrum protein mTagBFP2 (Subach *et al*., 2011). These developments have significantly broadened the application potential of fluorescent proteins to study various aspects of bacterial physiology.

A wide range of applications for fluorescent protein expression are available for common laboratory strains and pathogens, including *Escherichia coli* (Chalfie *et al*., 1994; Ma *et al*., 2011), *Salmonella* spp. (Ma *et al*., 2011; Robijns *et al*., 2014) and *Listeria* spp. (Ma *et al*., 2011; Van der Veen and Abee, 2011). However, fluorescent labelling of probiotic bacteria, known as ‘live microorganisms that, when administered in adequate amounts, confer a health benefit on the host’ (Hill *et al*., 2014), is less widespread and has long remained limited to traditional fluorescent proteins, such as GFP and its derivatives (Geoffroy *et al*., 2000; De Keersmaecker *et al*., 2006a; Mota *et al*., 2006; Van der Veen and Abee, 2011). Nevertheless, a wide variety of novel fluorescent proteins with advanced properties that have not yet been tested in probiotic strains, such as mTagBFP2, are currently available for heterologous expression in bacteria, which can offer additional benefits for the study of microbe–microbe and host–microbe interactions (Olenych *et al*., 2007; Dean and Palmer, 2014).

One important feature for many probiotics is the ability to prevent or treat the occurrence of pathogens in various human body niches and restore microbial homeostasis (Bron *et al*., 2011; Reid *et al*., 2011; Ritchie and Romanuk, 2012). For example, it has previously been shown that specific probiotic *Lactobacillus* strains and their molecular products are capable of inhibiting biofilms of *Salmonella* spp. (De Keersmaecker *et al*., 2006b; Petrova *et al*., 2016a), *Listeria monocytogenes*,* Pseudomonas aeruginosa* (Alexandre *et al*., 2014), *E. coli* O157:H7 (Gómez *et al*., 2016) and uropathogenic *E. coli* (UPEC) (Petrova *et al*., 2016a). In addition, certain probiotic strains are capable of affecting host immune responses (Karlsson *et al*., 2012; Lebeer *et al*., 2012), potentially promoting pathogen recognition and elimination and/or a decrease in inflammatory signalling. The ability of probiotic bacterial cells to adhere to the lining of their respective niche might enhance these beneficial effects. This can be explained by an increased retention time and possibly closer and more prolonged contact with the host epithelial and immune cells, although adherence is not an absolute prerequisite (Lebeer *et al*., 2008).

Prominent examples of widely used and well‐studied probiotic strains with demonstrated beneficial effects on the host are *L. rhamnosus* GG (a gastrointestinal isolate) (Segers and Lebeer, 2014) and *L. rhamnosus* GR‐1 (an urogenital isolate) (Reid *et al*., 1987). Nevertheless, understanding their mechanisms of action by which they improve and/or maintain human health remains a challenge. Therefore, in this study, we aimed to express various fluorescent proteins in the model strains *L. rhamnosus* GG and *L. rhamnosus* GR‐1. The recombinant strains that showed the highest count of fluorescent cells and strongest fluorescence compared to the wild‐type control strains were used to study their behaviour in mixed‐species biofilms with *Salmonella* Typhimurium–*gfpmut3*, as well as for interactions with human epithelial cells *in vitro*.

## Results

### Expression of mTagBFP2 and mCherry in *Lactobacillus rhamnosus* GG and *Lactobacillus rhamnosus* GR‐1 results in a fluorescent phenotype

The NICE system was implemented for the expression of fluorescent proteins in *L. rhamnosus* GG and *L. rhamnosus* GR‐1 under the control of the inducible *nisA* promoter, as it was previously optimized and successfully used for expression of *gfp* in *L. rhamnosus* GG (De Keersmaecker *et al*., 2006a). The pMEC45‐derived plasmids pCMPG11260, pCMPG11261, pCMPG11262 and pCMPG11263 containing respectively *mTagBFP2, mCherry, mVenus* or *ecfp* downstream of the *nisA* promoter (Fig. S1) were introduced by electroporation into the *nisRK‐*containing FAJ1905 strain of *L. rhamnosus* GG and the CMPG11259 strain of *L. rhamnosus* GR‐1 (Table 1).

**Table 1:** Bacterial strains and plasmids used in this study

Strain or plasmid	Genotype and/or phenotype	Source and/or references
Strain		
*E. coli* MC1061	araD139; Δ(ara, leu)7697; ΔlacX74; galU‐; gal; hsr−; hsm+; strA	Casadaban and Cohen (1980)
*Salmonella enterica* serovar	*S*. Typhimurium (ATCC 14028) variant carrying pFPV25.1 with *gfpmut3* for constitutive GFP production	Robijns *et al*. (2014)
Typhimurium (ATCC 14028) –*gfpmut3*		
*L. rhamnosus* GG (ATCC 53103)	Wild‐type human isolate	Kankainen *et al*. (2009)
*L. rhamnosus* GR‐1 (ATCC 5582)	Wild‐type human isolate	Reid *et al*. (2001)
FAJ1905	*L. rhamnosus* GG with chromosomal insertion of pMEC10 in *attB* (phage mv4); Ery^R^	De Keersmaecker *et al*. (2006a)
CMPG11259	*L. rhamnosus* GR‐1 with chromosomal insertion of pMEC10 in *attB* (phage mv4); Ery^R^	This study
CMPG5357	*L. rhamnosus* GG ΔspaCBA::Tc^R^ knockout mutant	Lebeer *et al*. (2012)
CMPG11260	FAJ1905 strain of *L. rhamnosus* GG carrying pCMPG11260 with *mTagBFP2*; Ery^R^, Cm^R^	This study
CMPG11261	FAJ1905 strain of *L. rhamnosus* GG carrying pCMPG11261 with *mCherry*; Ery^R^, Cm^R^	This study
CMPG11262	FAJ1905 strain of *L. rhamnosus* GG carrying pCMPG11262 with *mVenus*; Ery^R^, Cm^R^	This study
CMPG11263	FAJ1905 strain of *L. rhamnosus* GG carrying pCMPG11263 with *ecfp*; Ery^R^, Cm^R^	This study
FAJ1905/pMEC45	FAJ1905 carrying pMEC45 with *gfp;* Ery^R^, Cm^R^	De Keersmaecker *et al*. (2006a)
CMPG11269	CMPG5357 strain of *L. rhamnosus* GG carrying pCMPG11260 with *mTagBFP2*; Ery^R^, Cm^R^	This study
CMPG11270	CMPG5357 strain of *L. rhamnosus* GG carrying pCMPG11261 with *mCherry*; Ery^R^, Cm^R^	This study
CMPG11264	CMPG11259 strain of *L. rhamnosus* GR‐1 carrying pCMPG11260 with *mTagBFP2*; Ery^R^, Cm^R^	This study
CMPG11265	CMPG11259 strain of *L. rhamnosus* GR‐1 carrying pCMPG11261 with *mCherry*; Ery^R^, Cm^R^	This study
CMPG11266	CMPG11259 strain of *L. rhamnosus* GR‐1 carrying pCMPG11262 with *mVenus*; Ery^R^, Cm^R^	This study
CMPG11267	CMPG11259 strain of *L. rhamnosus* GR‐1 carrying pCMPG11263 with *ecfp*; Ery^R^, Cm^R^	This study
CMPG11268	CMPG11259 strain of *L. rhamnosus* GR‐1 carrying pMEC45 with *gfp;* Ery^R^, Cm^R^	
Plasmids		
pMEC10	Integration plasmid in attB at 3′ end of tRNAser locus (phage mv4); pNZ950‐derivative containing the 3′ end of *nisP* and *nisRK* (2.7 kb; *L. lactis* NZ9700) expressed by ery readthrough; Ery^R^	Pavan *et al*. (2000)
pMEC45	*L. lactis* pSH71 replicon; pNZ8037 derivative containing *gfp*_uv_ downstream of *nisA* promoter (*L. lactis* NZ9800); Cm^R^	Geoffroy *et al*. (2000)
pCMPG11260	Derivative of pMEC45 containing *mTagBFP2* cloned from pBAD‐*mTagBFP2* downstream of *nisA* promoter; Cm^R^	This study
pBAD‐*mTagBFP2*	Plasmid used for *mTagBFP2* amplification	Subach *et al*. (2011)
pCMPG11261	Derivative of pMEC45 containing *mCherry* cloned from pRSETb–*mCherry* downstream of *nisA* promoter; Cm^R^	This study
pRSETb–*mCherry*	Plasmid used for *mCherry* amplification	Shaner *et al*. (2004)
pCMPG11262	Derivative of pMEC45 containing *mVenus* cloned from pCMPG13918 downstream of *nisA* promoter; Cm^R^	This study
pCMPG13918	Plasmid used for *mVenus* amplification	Verstraeten *et al*., unpublished
pCMPG11263	Derivative of pMEC45 containing *ecfp* cloned from pECFP (Clontech) downstream of *nisA* promoter; Cm^R^	This study
pECFP	Plasmid used for *ecfp* amplification	Clontech

Cm^R^, chloramphenicol resistance; Ery^R^, erythromycin resistance; Tc^R^, tetracyclin resistance.

**Figure 1:**
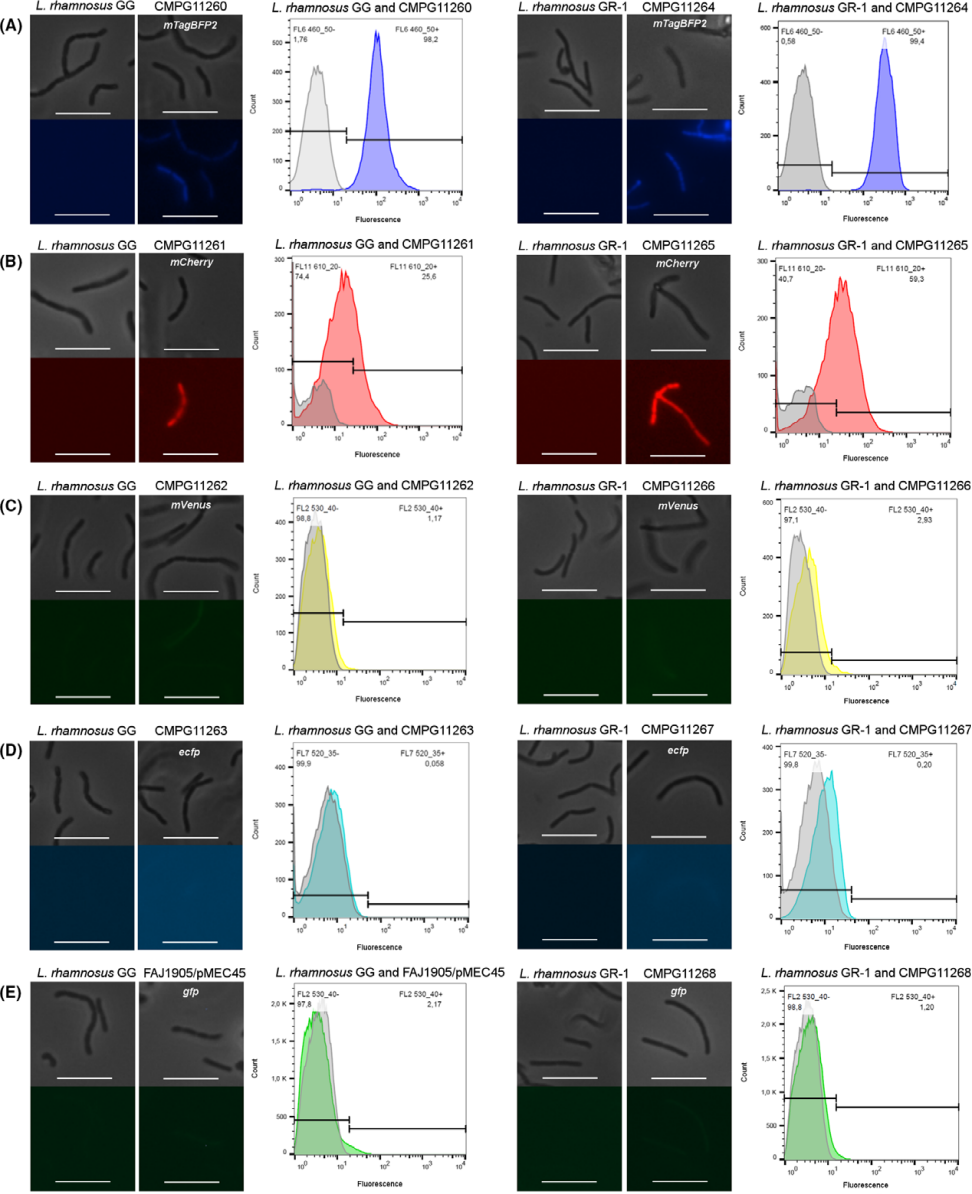
Detection of possible fluorescent phenotypes by epifluorescence microscopy and flow cytometric analysis of nisin‐induced *Lactobacillus rhamnosus* GG and *L. rhamnosus* GR‐1 bacterial cells implementing the NICE system to produce (A) mTagBFP2 (CMPG11260 and CMPG11264, in blue), (B) mCherry (CMPG11261 and CMPG11265, in red), (C) mVenus (CMPG11262 and CMPG11266, in yellow), (D) ECFP (CMPG11263 and CMPG11267, in cyan) and (E) GFP (FAJ1905/pMEC45 and CMPG11268, in green). In each panel, top photographs represent phase contrast, and bottom photographs represent fluorescent microscopy of the same field, with the white bars being equal to 10 μm. For each histogram, the fluorescence intensities at the corresponding wavelengths are indicated on the *x*‐axis, and the number of events/cell counts is indicated on the *y*‐axis. Wild‐type *L. rhamnosus* GG and *L. rhamnosus* GR‐1 were used as negative controls (depicted in grey).

One of the colonies showing the presence of the correct corresponding plasmid (as confirmed by PCR and sequencing) was selected for detection of fluorescent signals. Following induction with commercial nisin from *Lactococcus lactis*, a fluorescent phenotype could be observed with epifluorescence microscopy in CMPG11260 (*L. rhamnosus* GG carrying *mTagBFP2*), CMPG11261 (*L. rhamnosus* GG carrying *mCherry*), CMPG11264 (*L. rhamnosus* GR‐1 carrying *mTagBFP2*) and CMPG11265 (*L. rhamnosus* GR‐1 carrying *mCherry*) compared to the corresponding wild‐type *L. rhamnosus* GG or *L. rhamnosus* GR‐1 used as negative control (Fig. 1A and B). For the strains CMPG11262 (*L. rhamnosus* GG carrying *mVenus*), CMPG11263 (*L. rhamnosus* GG carrying *ecfp*), FAJ1905/pMEC45 (*L. rhamnosus* GG carrying *gfp*), CMPG11266 (*L. rhamnosus* GR‐1 carrying *mVenus*), CMPG11267 (*L. rhamnosus* GR‐1 carrying *ecfp*) and CMPG11268 (*L. rhamnosus* GR‐1 carrying *gfp*), no clear distinction at the expected wavelengths with the intrinsic fluorescence produced by the wild‐type *L. rhamnosus* GG and *L. rhamnosus* GR‐1 could be observed (Fig. 1C,D and E).

Subsequently, the fluorescence signals were quantified using flow cytometry. Fluorescent signals for the induced CMPG11260 (*L. rhamnosus* GG carrying *mTagBFP2*), CMPG11261 (*L. rhamnosus* GG carrying *mCherry*), CMPG11264 (*L. rhamnosus* GR‐1 carrying *mTagBFP2*) and CMPG11265 (*L. rhamnosus* GR‐1 carrying *mCherry*) strains were detected at the expected wavelength as compared to the corresponding wild‐type *L. rhamnosus* GG or *L. rhamnosus* GR‐1, which confirmed the microscopy results (Fig. 1A and B). CMPG11260 and CMPG11264 showed the most optimal detection of fluorescence when compared to the fluorescence signal of the wild‐type control strains, with respectively 98.2% and 99.4% of the analysed events classified as fluorescent (Fig. 1A). For CMPG11261 and CMPG11265, only 25.6% and 59.3% of cells respectively showed no overlap with the fluorescence signal of the respective wild‐type control strains when analysed with flow cytometry (Fig. 1B). No clear distinction in fluorescence compared with the respective wild‐type controls was observed for the strains CMPG11262 and CMPG11266 carrying *mVenus*, CMPG11263 and CMPG11267 carrying *ecfp*, and FAJ1905/pMEC45 and CMPG11268 carrying *gfp* (Fig. 1C,D and E).

**Figure 2:**
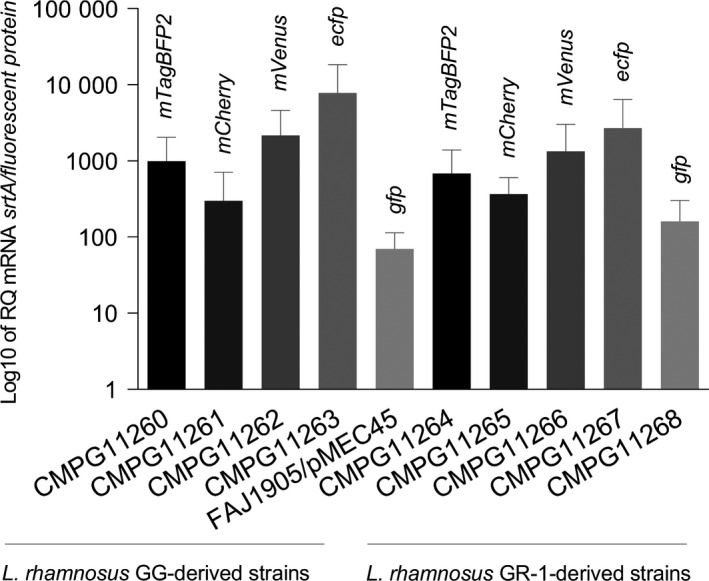
Fluorescent protein‐encoding gene expression in nisin‐induced *Lactobacillus rhamnosus* GG and *L. rhamnosus* GR‐1 strains quantified by qRT‐PCR. Data are presented as means with standard deviations indicating a ratio of the mRNA level for the fluorescent protein‐encoding genes over that of a housekeeping *srtA* gene.

To explore whether the lack of fluorescence in the recombinant *L. rhamnosus* GG and *L. rhamnosus* GR‐1 strains containing *mVenus, ecfp* and *gfp* was due to inefficient gene expression, a real‐time quantitative PCR (qRT‐PCR) analysis was performed on all the strains designed to express *mTagBFP2, mCherry, mVenus, ecfp* and *gfp* using the NICE system. qRT‐PCR analysis for mRNA levels of expressed genes demonstrated that all of the fluorescent protein‐encoding genes were expressed, although the amount of detected mRNA varied between the tested strains and the respective fluorescent protein genes (Fig. 2). Even though no fluorescence was observed for the strains designed to express *ecfp* and *mVenus* compared with the wild‐type negative controls, high levels of both *ecfp* and *mVenus* mRNA could be detected in CMPG11262 and CMPG11266, and CMPG11263 and CMPG11267 respectively (Fig. 2).

**Figure 3:**
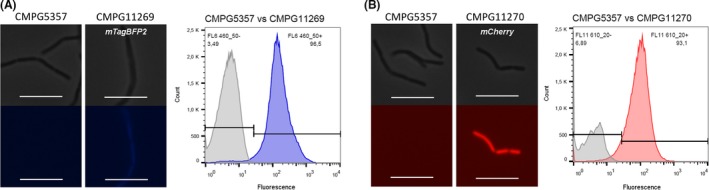
Detection of a fluorescent phenotype by epifluorescence microscopy and flow cytometric analysis of nisin‐induced CMPG5357, a *Lactobacillus rhamnosus* GG pili knockout mutant, implementing the NICE system to produce mTagBFP2 (strain CMPG11269, A, in blue) and mCherry (strain CMPG11270, B, in red). In each panel, top photographs represent phase contrast, and bottom photographs represent fluorescent microscopy of the same field. The white bar is equal to 10 μm. For each histogram, the fluorescence intensities at the corresponding wavelengths are indicated on the *x*‐axis, and the number of events/cell counts is indicated on the *y*‐axis. CMPG5357 was used as negative control (depicted in grey).

The NICE system was likewise implemented to fluorescently label the CMPG5357 strain, a *L. rhamnosus* GG Δ*spaCBA*::Tc^R^ knockout mutant previously developed by our group to study the functional role of SpaCBA pili in interactions with the host (Lebeer *et al*., 2012). The pCMPG11260 and pCMPG11261 plasmids for the expression of respectively *mTagBFP2* and *mCherry* under the control of the *nisA* promoter were introduced into CMPG5357 by electroporation, resulting in CMPG11269 and CMPG11270. The presence of plasmids with the correct insert was confirmed by PCR and plating out as described above for the wild‐type recombinant strains. A fluorescent phenotype following induction with nisin could be observed in both CMPG11269 (CMPG5357 carrying *mTagBFP2*) and CMPG11270 (CMPG5357 carrying *mCherry*). The fluorescent signals at the expected wavelengths were visually confirmed by epifluorescence microscopy and quantified using flow cytometric analysis that classified 96.5% of CMPG11269 cells (Fig. 3, in blue) and 93.1% of CMPG11270 cells as fluorescent (Fig. 3, in red).

In addition to the *nisA* promoter of the NICE system, the use of constitutive or internally regulated promoters for the *msp1*,* msp2*,* spaCBA*,* welE* genes and *dlt* operon from *L. rhamnosus* combined with an integrating pEM40 plasmid has been analysed for fluorescent protein expression in the context of this study. However, up to now, none of the constitutive promoters resulted in a detectable fluorescence signal in *L. rhamnosus* under the tested conditions.

### Visualization of mixed‐species biofilms of fluorescent *Lactobacillus rhamnosus* and *Salmonella* Typhimurium strains

**Figure 4:**
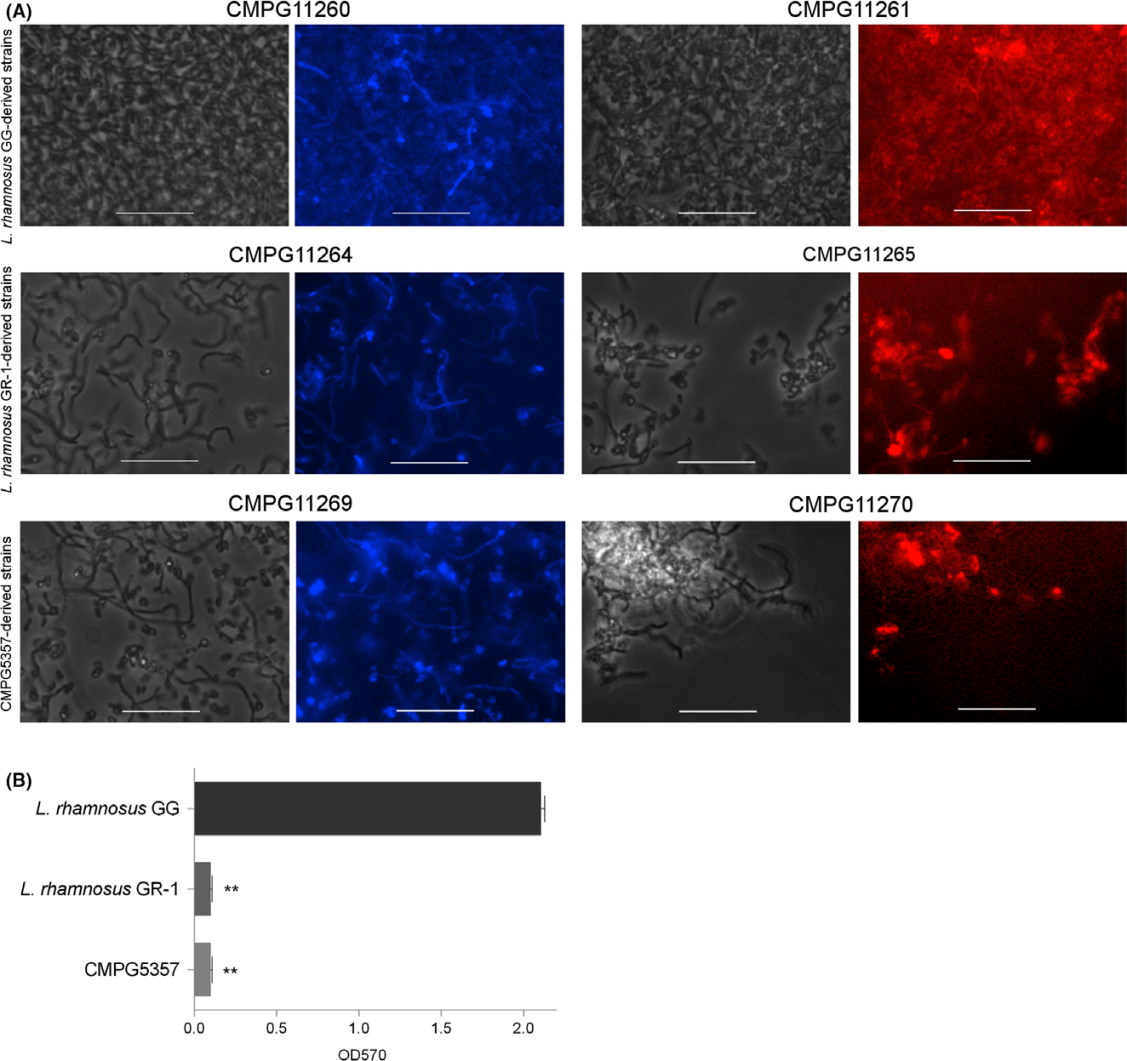
Single‐strain biofilms of nisin‐induced fluorescent *Lactobacillus rhamnosus* strains after 48 h incubation in AOAC medium.(A) Biofilms of CMPG11260, an *L. rhamnosus* GG strain producing mTagBFP2 (phase contrast in grey and fluorescent in blue), CMPG11264, an *L. rhamnosus* GR‐1 strain producing mTagBFP2 (phase contrast in grey and fluorescent in blue), CMPG11269, the CMPG5357 strain producing mTagBFP2 (phase contrast in grey and fluorescent in blue), CMPG11261, an *L. rhamnosus* GG strain producing mCherry (phase contrast in grey and fluorescent in red), CMPG11265, an *L. rhamnosus* GR‐1 strain producing mCherry (phase contrast in grey and fluorescent in red) and CMPG11270, the CMPG5357 strain producing mCherry (phase contrast in grey and fluorescent in red) are shown. White bars are equal to 20 μm. (B) Bar graph depicting biofilm formation on pegs by wild‐type *L. rhamnosus* GG,* L. rhamnosus* GR‐1 and the CMPG5357 pili knockout mutant of *L. rhamnosus* GG in AOAC. Data are presented as means with standard deviations. Statistically significant differences of *P* ≤ 0.01 between wild‐type *L. rhamnosus* GG and *L. rhamnosus* GR‐1 or CMPG5357 are depicted with double asterisks.

**Figure 5:**
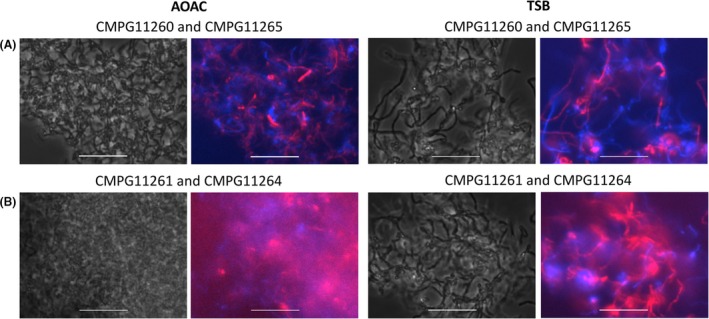
Mixed *Lactobacillus* strains biofilms of nisin‐induced fluorescent *Lactobacillus rhamnosus* strains after 48 h co‐incubation in AOAC or TSB media. (A) Biofilms of *L. rhamnosus* GG‐derived CMPG11260 producing mTagBFP2 (phase contrast in grey and fluorescent in blue) with *L. rhamnosus* GR‐1‐derived CMPG11265 producing mCherry (phase contrast in grey and fluorescent in red). (B) Biofilms of *L. rhamnosus* GG‐derived CMPG11261 producing mCherry (phase contrast in grey and fluorescent in red) with *L. rhamnosus* GR‐1‐derived CMPG11264 producing mTagBFP2 (phase contrast in grey and fluorescent in blue). White bars are equal to 20 μm.

Biofilm formation by single strains (Fig. 4) and mixed strains (Fig. 5) of fluorescent *L. rhamnosus* was explored using visualization with epifluorescence microscopy. CMPG11260 and CMPG11261, both *L. rhamnosus* GG wild‐type‐derived strains (Fig. 4A, CMPG11260 in blue and CMPG11261 in red), showed formation of thick biofilms in AOAC. Biofilm thickness was clearly reduced for CMPG11269 and CMPG11270, derived from the pili knockout mutant of *L. rhamnosus* GG (Fig. 4A, CMPG11269 in blue and CMPG11270 in red), in agreement with our previous work showing the key role of pili in biofilm formation (Lebeer *et al*., 2012). Lower levels of biofilm formation in AOAC medium were also observed for CMPG11264 and CMPG11265, both *L. rhamnosus* GR‐1‐derived strains (Fig. 4A, CMPG11264 in blue and CMPG11265 in red). These epifluorescence microscopy observations correspond with the results obtained from the Calgary biofilm formation experiments of the wild‐type *L. rhamnosus* GG, *L. rhamnosus* GR‐1 and the CMPG5357 pili knockout mutant of *L. rhamnosus* GG (Fig. 4B).

Fluorescent *L. rhamnosus* cells can also be distinguished from each other in mixed‐strain lactobacilli biofilm set‐ups in AOAC or TSB, such as those of CMPG11260 (Fig. 5A, in blue) with CMPG11265 (Fig. 5A, in red), as well as CMPG11261 (Fig. 5B, in red) with CMPG11264 (Fig. 5B, in blue). The *L. rhamnosus* GG‐derived strains CMPG11260 and CMPG11261 demonstrate a more prominent biofilm formation capacity compared to CMPG11264 and CMPG11265 (Fig. 5), confirming the results from the single‐strain biofilm formation experiment (Fig. 4). However, intriguingly, the *L. rhamnosus* GR‐1‐derived strains CMPG11264 and CMPG11265 also showed biofilm formation when co‐cultured with the *L. rhamnosus* GG‐derived CMPG11260 and CMPG11261 strains (Fig. 5).

**Figure 6:**
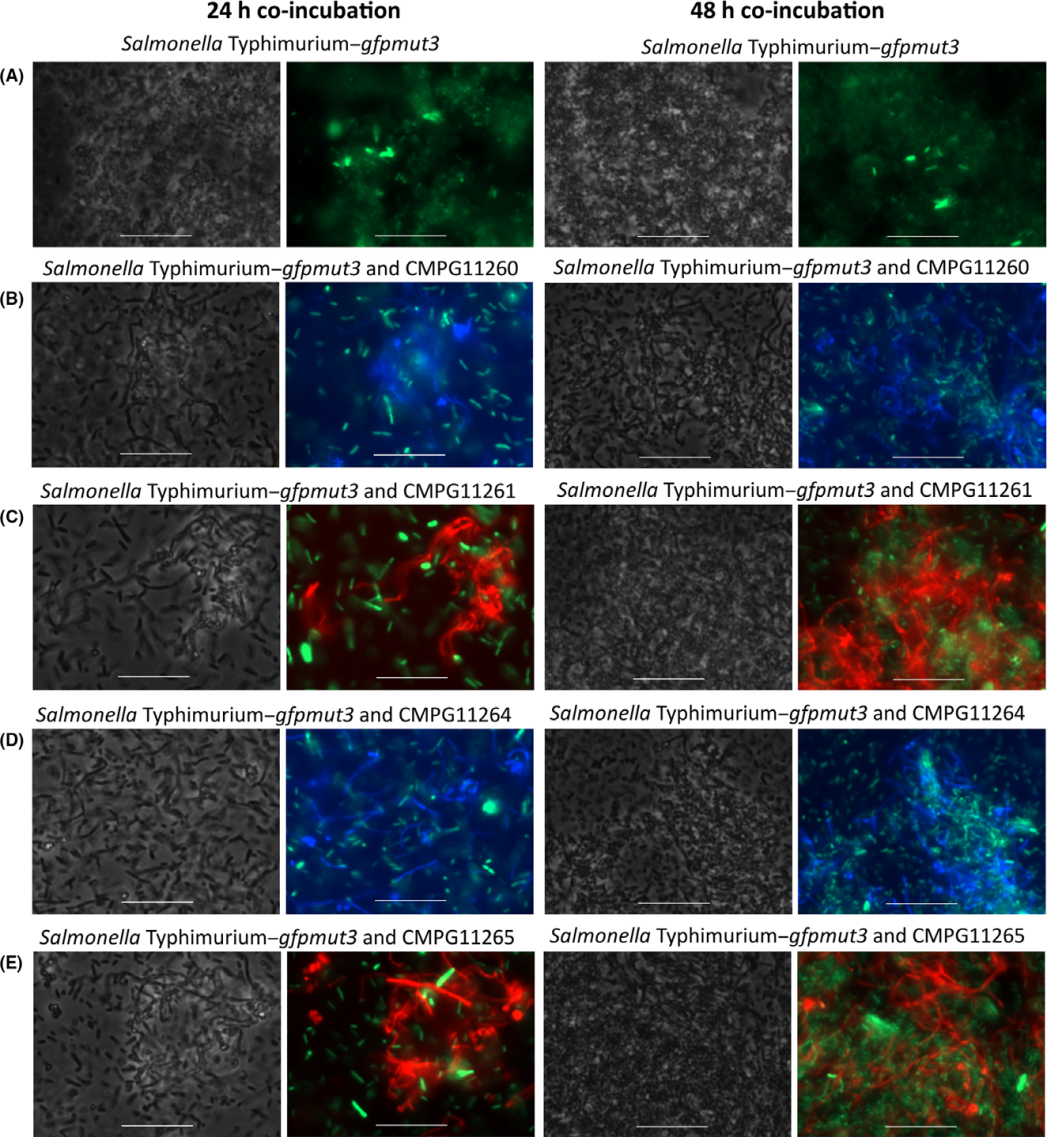
Mixed‐species biofilms of *gfp*‐expressing *S*. Typhimurium–*gfpmut3* and nisin‐induced fluorescent *Lactobacillus rhamnosus* strains after 24 h (left set of panels) and 48 h (right set of panels) co‐incubation. *S*. Typhimurium–*gfpmut3* was incubated alone (A) (phase contrast in grey and fluorescent in green in all panels) or co‐incubated with CMPG11260 producing mTagBFP2 (B) (phase contrast in grey and fluorescent in blue), CMPG11261 producing mCherry (C) (phase contrast in grey and fluorescent in red), CMPG11264 producing mTagBFP2 (D) (phase contrast in grey and fluorescent in blue) or CMPG11265 producing mCherry (E) (phase contrast in grey and fluorescent in red). White bars are equal to 20 μm.

To validate the functionality of the *L. rhamnosus* strains successfully expressing *mCherry* and *mTagBFP2*, microbe–microbe interactions in mixed‐species biofilms with fluorescent *S*. Typhimurium–*gfpmut3* were studied. A clear visual distinction could be made between the *gfp*‐expressing *S*. Typhimurium–*gfpmut3* cells (Fig. 6A–E, in green) and the induced recombinant *L. rhamnosus* cells CMPG11260 (Fig. 6B, in blue), CMPG11261 (Fig. 6C, in red), CMPG11264 (Fig. 6D, in blue) and CMPG11265 (Fig. 6E, in red). Fluorescence of induced recombinant *L. rhamnosus* cells could still be observed after 24 h (Fig. 6, left set of panels) and after 48 h (Fig. 6, right set of panels) of co‐incubation without additional induction with nisin. In these co‐interaction experiments, biofilm formation by *S*. Typhimurium–*gfpmut3* was reduced by the lactobacilli compared to the negative control *S*. Typhimurium–*gfpmut3* alone in all tested conditions (Fig. 6B–E compared to A).

### Fluorescent *Lactobacillus rhamnosus* can be used to detect adhesion to human epithelial cells *in vitro*

**Figure 7:**
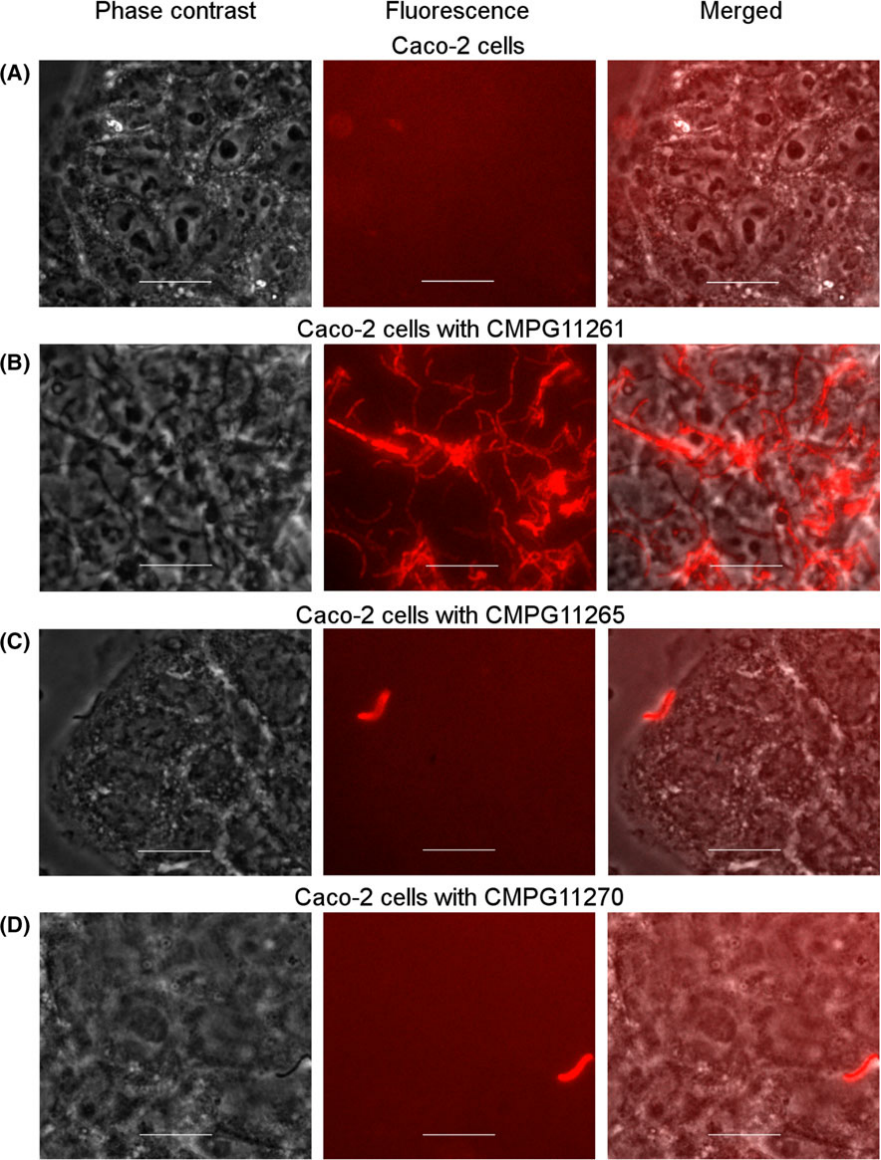
Adhesion pattern of *Lactobacillus rhamnosus* strains expressing *mCherry* to intestinal epithelial Caco‐2 cells visualized by epifluorescence microscopy. (A) Caco‐2 cells alone used as negative control; (B) Caco‐2 cells incubated with CMPG11261 (*L. rhamnosus* GG expressing mCherry, in red); (C) Caco‐2 cells incubated with CMPG11265 (*L. rhamnosus* GR‐1 expressing mCherry, in red); (D) Caco‐2 cells incubated with CMPG11270 (CMPG5357 *spaCBA* knockout expressing mCherry, in red). In each set of panels, left panel represent phase contrast, middle panel represents fluorescence microscopy and right panel represents a combination of phase contrast with fluorescent microscopy. White bars are equal to 20 μm.

To further validate the potential use of fluorescent *L. rhamnosus* strains in unravelling probiotic interactions with the host, an adhesion assay to human intestinal epithelial Caco‐2 cells was conducted. The adhesion capacities of *L. rhamnosus* GG expressing *mCherry* (CMPG11261, Fig. 7B), *L. rhamnosus* GR‐1 expressing *mCherry* (CMPG11265, Fig. 7C) and the CMPG5357 *spaCBA* pili knockout mutant of *L. rhamnosus* GG expressing *mCherry* (CMPG11270, Fig. 7D) to Caco‐2 cells were compared. Only the *mCherry*‐expressing strains could be visualized by epifluorescence microscopy with Caco‐2 cell cultures, as the fluorescence produced by the *mTagBFP2*‐expressing strains could not be distinguished from the intrinsic background fluorescence of the cell cultures at the corresponding wavelength. The *L. rhamnosus* GG‐derived strain CMPG11261 showed a significant level of interaction with the cells and could be visualized in large numbers on top of the Caco‐2 cell layer (Fig. 7B). In comparison, the *L. rhamnosus* GR‐1‐derived strain CMPG11265 (Fig. 7C) demonstrated significantly less binding to the Caco‐2 cell layer. Likewise, the *L. rhamnosus* GG pili knockout mutant‐derived strain CMPG11270 (Fig. 7D) demonstrated a drastically diminished adhesion capacity to Caco‐2 cells compared with the *L. rhamnosus* GG‐derived strain CMPG11261 (Fig. 7B), as it has previously been reported that SpaCBA is the key adhesin promoting the good adhesion capacities of *L. rhamnosus* GG to human intestinal epithelial cells (Lebeer *et al*., 2012).

## Discussion

In this study, we developed novel fluorescently labelled probiotic strains to study their interactions with other microorganisms, as well as the host. These strains can be used to better understand probiotic mechanisms of action, as bacterial fluorescence is a powerful tool to visualize and quantify the probiotic cells. The repertoire of fluorescent proteins expressed in probiotic lactobacilli is currently limited, and its expansion remains potentially challenging due to the microbiological properties of lactic acid bacteria, such as the low pH of the bacterial cytoplasm and culture medium, anaerobic growth conditions and the specific environments of the niche in which the strain is studied.

Here, we demonstrate successful labelling of wild‐type and mutant strains of the commercially available probiotics *L. rhamnosus* GG and *L. rhamnosus* GR‐1 by heterologous expression of the mTagBFP2 and mCherry fluorescent proteins. To our knowledge, this is the first report of the monomeric blue protein mTagBFP2 being used to label any probiotic *Lactobacillus* strain. Furthermore, no heterologous expression of fluorescent proteins in the model vaginal probiotic strain *L. rhamnosus* GR‐1 has been described to date. Significant fluorescence could be demonstrated in the novel recombinant *L. rhamnosus* strains when the mTagBFP2 and mCherry proteins were expressed using the nisin‐inducible expression system (NICE) system, while this was not observed for the strains designed to express the mVenus or ECFP proteins. The maturation of traditionally used fluorescent proteins, such as GFP, and some of its derivatives, such as GFPuv, mVenus and ECFP, can be especially sensitive to changes in pH, low oxygen availability and growth temperatures (Scott *et al*., 2000; Olenych *et al*., 2007). For example, it has been demonstrated that increased aeration and artificial buffering of the growth medium pH are required for the optimal production and fluorescence of GFP in *L. casei* (Pérez‐Arellano and Pérez‐Martínez, 2003). Certain drawbacks resulting from the combination of fluorescent proteins with the properties of specific strains can be compensated for using a superior gene expression system that results in higher protein yield, such as the NICE system (Geoffroy *et al*., 2000; De Keersmaecker *et al*., 2006a). The NICE system is potentially food grade and offers high protein expression levels, although it presents a number of limitations, including the requirement for nisin induction and a dual plasmid composition (Geoffroy *et al*., 2000). To overcome these limitations, the use of constitutive or internally regulated promoters might be considered. However, until now, we were unable to obtain a detectable fluorescence signal when using constitutive promoters in *L. rhamnosus* under the tested conditions; therefore, the NICE system was chosen for further experiments. Nevertheless, even when the heterologous protein expression system is effective, the resulting fluorescence will still largely depend on the choice of a specific fluorescent protein and its maturation properties. For example, even under the optimized conditions, only 55% of the GFP produced in *L. casei* was in fact fluorescent (Pérez‐Arellano and Pérez‐Martínez, 2003). As demonstrated in this study for the strains carrying *mVenus* and *ecfp*, efficient fluorescent protein gene expression indeed does not always result in significant fluorescence in lactobacilli, even when corresponding mRNA production can be demonstrated. This might be the result of potential protein aggregation or impaired folding or maturation of proteins such as mVenus and ECFP in the cytoplasmic conditions of *L. rhamnosus*, which could impair the development of fluorescence even when sufficient mRNA production and translation occur. In addition, codon optimization of fluorescent protein genes could be considered (Van der Veen and Abee, 2011; Karimi *et al*., 2016) to potentially improve fluorescent protein translation and folding and the resulting fluorescence in lactobacilli. Although fluorescent protein mRNA production for each strain could be demonstrated, comparative analysis of mRNA levels corresponding to different fluorescent proteins is complicated due to the use of different qRT‐PCR primer couples, which could affect amplification efficiency.

A number of recent studies have shown that fluorescent proteins other than GFP and its derivatives can indeed be useful for investigating *Lactobacillus* and *Bifidobacterium* strains in certain *in vivo* and *in vitro* assays (Russo *et al*., 2015; Karimi *et al*., 2016). In our study, we used mTagBFP2 and mCherry, as they are among the most promising candidates for labelling probiotic bacteria due to their fast and efficient maturation at 37°C, high photostability and lower pH sensitivity compared to GFP and its derivatives (Olenych *et al*., 2007). mCherry has recently been used with success for labelling other probiotic *Lactobacillus* strains, such as *L. reuteri* (Karimi *et al*., 2016), *L. plantarum* and *L. fermentum* (Russo *et al*., 2015), and it also performed well in this study. Indeed, the majority of induced cells designed to express the mCherry protein demonstrated a fluorescent phenotype when analysed by flow cytometry. This was even more pronounced for the *L. rhamnosus* strains producing mTagBFP2, as 95% or more cells designed to express *mTagBFP2* were classified as fluorescent. Furthermore, for both mTagBFP2 and mCherry, we could show a persistence of fluorescence in recombinant *L. rhamnosus* strains even 48 h after induction with nisin, which makes these strains especially useful for long‐term *in vitro* and potentially *in vivo* studies.

Tagging probiotic strains not only with the commonly used GFP, but also with the more recently developed variants of fluorescent proteins, such as mCherry and mTagBFP2, allows for an expanded spectral range and improved bacterial visualization. In this study, we demonstrate that fluorescent *L. rhamnosus* strains can be visualized in mixed‐species biofilms, as bacterial cells of GFP‐labelled *S*. Typhimurium and different mCherry‐ and mTagBFP2‐producing *L. rhamnosus* strains can be distinguished from each other based on their fluorescent tagging. This is especially useful for unravelling probiotic mechanisms of action against pathogenic microorganisms, as many bacterial pathogens, including *L. monocytogenes* (Gómez *et al*., 2016), uropathogenic *E. coli* (Petrova *et al*., 2016a) and *P. aeruginosa* (Alexandre *et al*., 2014), are capable of forming biofilms that contribute to their enhanced survival, tolerance and spreading (Olson *et al*., 2002; Hobley *et al*., 2015; Steenackers *et al*., 2016). In this study, visualization of mixed‐species biofilms by epifluorescence microscopy demonstrated an inhibitory effect of *L. rhamnosus* GG and *L. rhamnosus* GR‐1 on biofilm formation by *S*. Typhimurium. This is in line with previous studies describing a strong antimicrobial effect of *L. rhamnosus* GG and its molecular products on *S*. Typhimurium viability (Hutt *et al*., 2006; Marianelli *et al*., 2010) and biofilm formation (De Keersmaecker *et al*., 2006b; Petrova *et al*., 2016a). Therefore, these probiotic strains can potentially be used for prevention of *S*. Typhimurium infections and biofilm formation in human and animal disease and food applications. In future studies, a similar approach can be used for testing probiotic interactions with other biofilm‐forming microorganisms, such as the clinically relevant strains of *E. coli, Clostridium difficile* or other pathogens commonly found in the gastrointestinal and vaginal niches.

In addition, visual analysis of single strain and mixed‐strain biofilms of different fluorescent strains of *L. rhamnosus* has demonstrated a diminished capacity for biofilm formation by *L. rhamnosus* GR‐1 and the CMPG5357 *spaCBA* knockout mutant strain of *L. rhamnosus* GG compared to *L. rhamnosus* GG. It has previously been demonstrated that *L. rhamnosus* GG is capable of efficient biofilm formation *in vitro* (Lebeer *et al*., 2007). Our results support the previously developed notion that the SpaCBA pili play a crucial role in this process (Lebeer *et al*., 2012), as both *L. rhamnosus* GR‐1 and the *spaCBA* knockout mutant strain lack the pili structures. Surprisingly, when *L. rhamnosus* GR‐1 is co‐incubated with *L. rhamnosus* GG, its biofilm formation properties are enhanced compared to its single‐strain biofilms, possibly due to the embedding of *L. rhamnosus* GR‐1 into the *L. rhamnosus* GG biofilm matrix. Therefore, expression of fluorescent proteins in both pathogens (Ma *et al*., 2011; Robijns *et al*., 2014) and probiotic bacteria (Van der Veen and Abee, 2011) provides a non‐destructive labelling method for real‐time observation of biofilm formation dynamics and mixed‐species biofilm interactions.

Furthermore, in this study, we show that the fluorescent *L. rhamnosus* strains, in particular those expressing the red mCherry fluorescent protein, can serve as a useful tool in studying probiotic interactions with human host cells. The adhesion pattern to intestinal epithelial Caco‐2 cells visualized with epifluorescence microscopy confirms high binding capacity of *L. rhamnosus* GG to the intestinal epithelium, which is one of the hallmark characteristics of this probiotic strain (Segers and Lebeer, 2014). Results previously obtained by our group suggest that SpaCBA pili present on the outer surface of *L. rhamnosus* GG cells play a key role in its adhesion to both intestinal epithelial cells (Lebeer *et al*., 2012) and murine macrophages (Vargas García *et al*., 2015). Therefore, SpaCBA pili might contribute to the longer retention time of *L. rhamnosus* GG in the human gastrointestinal tract compared with other *Lactobacillus* strains that lack pili‐like structures (Kankainen *et al*., 2009). Indeed, in our adhesion experiment, the non‐piliated *L. rhamnosus* GR‐1 strain expressing *mCherry* failed to demonstrate significant adhesion to Caco‐2 cells in the tested conditions. Likewise, a *spaCBA* knockout mutant of *L. rhamnosus* GG expressing *mCherry* showed a drastically diminished binding to Caco‐2 cells, further confirming our previous data (Lebeer *et al*., 2012).

In the present work, we demonstrate the implementation and feasibility of mTagBFP2 and mCherry fluorescent tagging of probiotic lactobacilli, thus further expanding the tools for studying their molecular and cellular probiotic interactions with other microorganisms, as well as the host. Due to the easily detectable fluorescence of individual cells that persists long after induction, these novel fluorescent strains demonstrate a strong application potential for both *in vitro* and animal *in vivo* probiotic experiments.

## Experimental procedures

### Bacterial strains, plasmids and growth conditions

The bacterial strains and plasmids used in this study are listed in Table 1. All *L. rhamnosus* strains were grown in Man–Rogosa–Sharpe (MRS) medium (Difco) at 37°C in static conditions resulting in a limited supply of oxygen. *Escherichia coli* MC1061 and *S*. Typhimurium–*gfpmut3* strains were grown at 37°C in Luria‐Bertani broth in shaking conditions at 200 r.p.m. When appropriate erythromycin and chloramphenicol were supplied at respectively 5 and 10 μg ml^−1^ for the *L. rhamnosus* strains, chloramphenicol was supplied at 10 μg ml^−1^ for *E. coli* MC1061 and ampicillin was supplied at 100 μg ml^−1^ for *S*. Typhimurium–*gfpmut3*. Production of fluorescent proteins by *L. rhamnosus* strains was induced with nisin as previously described with minor modifications (De Keersmaecker *et al*., 2006a). Briefly, an overnight culture was diluted 1:50 in prewarmed MRS and incubated for 30 min. The diluted culture was subsequently induced with commercial nisin from *Lactococcus lactis* (Sigma‐Aldrich, Diegem, Belgium, N5764) at a final concentration of 500 ng ml^−1^ and further incubated for 24 h.

### Construction of expression vectors and recombinant *Lactobacillus rhamnosus* strains

Standard molecular techniques were used for the construction of overexpression plasmids (Table 1), preparation of competent *E. coli* cells and transformation (Sambrook *et al*., 1989). Gibson assembly (Gibson *et al*., 2009) was additionally used for the construction of pCMPG11261 due to a NcoI restriction site present in the coding sequence of *mCherry*, which complicated its insertion in the NcoI cloning site of pMEC45 (Fig. S1) using traditional cloning methods. PCR primers were purchased from Integrated DNA Technologies (IDT) (Belgium) (Table S1). Enzymes used for PCR, restriction digests, dephosphorylation and ligation of constructs were purchased from New England Biolabs (Bioké, Leiden, the Netherlands) and used according to the manufacturer's instructions. Plasmid DNA was isolated from *E. coli* using QIAGEN miniprep kits (Qiagen Benelux, Antwerp, Belgium) and transferred to highly competent *L. rhamnosus* GG, *L. rhamnosus* GR‐1, CMPG5357, FAJ1905 or CMPG11259 cells by electroporation as described before (De Keersmaecker *et al*., 2006a; Petrova *et al*., 2016b). Electroporated *L. rhamnosus* cells were incubated at 37°C on MRS agar with corresponding antibiotics, *i.e*. erythromycin and chloramphenicol at respectively 5 and 10 μg ml^−1^ for strains electroporated with pMEC45‐derived constructs. The presence of plasmids with the correct insert in the resulting recombinant *L. rhamnosus* strains was confirmed by PCR analysis of colonies grown on erythromycin‐ and chloramphenicol‐containing MRS agar using the Pro‐352 forward primer and the respective reverse primers for the corresponding fluorescent protein genes, being S&P‐01367 for *mTagBFP2*, S&P‐01303 for *mCherry*, S&P‐01364 for *mVenus* and S&P‐01365 for *ecfp* (Table S1).

### Visualization of cell fluorescence with epifluorescence microscopy

Epifluorescence microscopy was performed using the Zeiss Axio Imager Z1 microscope with an EC Plan Neofluar (×40 magnification/0.3 numerical aperture) objective using the settings corresponding to the excitation and emission wavelengths of each expressed fluorescent protein. Images were obtained in aerobic conditions with an axiocam mrm and the axiovision software. Each visualization experiment was repeated at least twice.

### Quantification of bacterial cell fluorescence using flow cytometry

Flow cytometric analysis of *L. rhamnosus* strains carrying genes for fluorescent protein expression was performed using 24 h cultures incubated at 37°C with appropriate antibiotics and when necessary induced with nisin as previously described (De Keersmaecker *et al*., 2006a). Cultures were washed once and resuspended in PBS. Bacterial cell fluorescence was analysed in aerobic conditions by flow cytometry with a BD Influx cell sorter (Becton, Dickinson, Erembodegem, Belgium). Data were analysed using flowjo software (7.6.4), (FlowJo, LCC, Ashland, OR, USA), and histogram plots were obtained based on cell counts versus fluorescent signals at the expected wavelengths. Each flow cytometry quantification experiment was repeated at least twice.

### Quantification of fluorescent protein gene expression by qRT‐PCR

Total bacterial RNA was isolated using the SV Total RNA Isolation System Kit (Promega, Leiden, Netherlands) from 24 h cultures of *L. rhamnosus* strains incubated at 37°C with appropriate antibiotics and induced with nisin as previously described (De Keersmaecker *et al*., 2006a). To determine fluorescent protein gene expression, reverse transcription (RevertAid First Strand cDNA Synthesis Kit; Life Technologies Europe, Invitrogen, Gent, Belgium) with subsequent real‐time quantitative PCR (qRT‐PCR) (Power SYBR Green PCR Master Mix; Applied Biosystems Europe, Halle, Belgium) was performed. Primers were designed and synthetized by Integrated DNA Technologies (IDT) (Belgium) and are listed in Table S1. Relative abundance of mRNA corresponding to each fluorescent protein gene in relation to the housekeeping SrtA sortase mRNA was quantified after 40 amplification cycles of 15 s at 94°C and 1 min at 60°C. Expression plasmids containing each fluorescent protein‐encoding gene were used as cDNA plasmid controls, and mRNA from wild‐type *L. rhamnosus* GG and *L. rhamnosus* GR‐1 served as negative controls for each pair of primers.

### Co‐incubation in mixed‐species biofilm with *Salmonella* Typhimurium–gfpmut3

For the visualization of mixed‐species biofilms, a *S*. Typhimurium ATCC14028 strain constitutively expressing the *gfpmut3* gene was used, combined with the CMPG11260, CMPG11261, CMPG11264 or CMPG11265 fluorescent strains of *L. rhamnosus*.

To prepare fluorescent *L. rhamnosus* cells for the mixed‐species biofilm visualization experiments, overnight cultures of CMPG11260, CMPG11261, CMPG11264 or CMPG11265 were first induced with nisin as previously described (De Keersmaecker *et al*., 2006a) and incubated for 24 h before being diluted for biofilm formation. For the formation of 24 and 48 h mixed‐species biofilms with *S*. Typhimurium–*gfpmut3,* overnight cultures of planktonic *S*. Typhimurium–*gfpmut3* were diluted 1:100 in 1/20 TSB in plastic Petri dishes. Then, the previously induced 24 h cultures of CMPG11260, CMPG11261, CMPG11264 or CMPG11265 were immediately added at a 1:100 dilution to the Petri dishes already containing diluted *S*. Typhimurium–*gfpmut3* in 1/20 TSB. After co‐incubation of *S*. Typhimurium–*gfpmut3* with CMPG11260, CMPG11261, CMPG11264 or CMPG11265 for 24 or 48 h at 25°C in static aerobic conditions, the mixed‐species biofilms were visualized with epifluorescence microscopy.

For the biofilm visualization experiments of single strain or mixed strains fluorescent *L. rhamnosus* biofilm formation, overnight cultures of CMPG11260, CMPG11261, CMPG11264, CMPG11265, CMPG11269 or CMPG11270 were induced with nisin as previously described (De Keersmaecker *et al*., 2006a) and incubated for 24 h in MRS before being used for biofilm formation. The cultures were subsequently diluted 1:100 in 1/20 TSB or AOAC (Difco) medium in Petri dishes, and incubated at 25°C for 48 h in static aerobic conditions. The biofilms were visualized with epifluorescence microscopy.

Epifluorescence microscopy was performed using the Zeiss Axio Imager Z1 microscope with an EC Plan Neofluar (×40 magnification/0.3 numerical aperture). Images were obtained with an axiocam MRm and the axiovision software (ZEISS, Jena, Germany). Each visualization experiment was repeated at least twice.

For the quantification of biofilm formation, biofilms of *L. rhamnosus* GG, *L. rhamnosus* GR‐1 or CMPG5357 strains were grown on pegs in 96‐well plates for 72 h at 37°C in AOAC or TSB medium, and biofilm formation was evaluated using crystal violet staining as previously described (Lebeer *et al*., 2007). Sterile growth medium was included as negative control. Analysis for each of the strains was performed eight times. Statistical analysis of the data using the Kruskal–Wallis test and Dunn's multiple comparison test was performed in GraphPad Prism 5.

### *In vitro* assay for adhesion to human intestinal epithelial Caco‐2 cells

The human intestinal epithelial Caco‐2 cell line (American Type Culture Collection; Rockville, MD, USA; ATCC HTB‐37TM) was maintained in Dulbecco's modified Eagle's medium (DMEM)–F‐12 (Invitrogen) supplemented with 10% foetal calf serum (FCS) at 37°C, 5% CO_2_ and 90% relative humidity. For the visualization of *L. rhamnosus* adhesion to Caco‐2 cells, the cell cultures that had reached 70%–80% confluence were treated with trypsin and distributed over glass coverslips as previously described (Petrova *et al*., 2016a). Briefly, after 24 h of incubation in DMEM‐F‐12‐FCS at 37°C, 5% CO_2_ and 90% relative humidity, the cells were fixed on 13 mm coverslips in fixation buffer containing 0.05% glutaraldehyde and 2.5% formaldehyde for 1 h at 37°C, 5% CO_2_ and 90% relative humidity. Simultaneously, 10^7^ CFU of fluorescent *L. rhamnosus* cultures previously induced with nisin as described above were collected by centrifugation for 10 min at 2000 *g*, washed once and dissolved in 1× PBS, which was then added to the Caco‐2 cells. After 1 h co‐incubation at 37°C in aerobic conditions, the coverslips were washed twice in PBS to remove unattached bacterial cells and subsequently visualized by epifluorescence microscopy using the Zeiss Axio Imager Z1 microscope with an EC Plan Neofluar (×40 magnification/0.3 numerical aperture). Images were obtained with an AxioCam MRm and the AxioVision software. Each visualization experiment was repeated at least twice.

## Author's contribution

Irina Spacova, Sarah Lebeer and Mariya Petrova designed the experiments and wrote the manuscript. Irina Spacova, Mariya Petrova, Tine Verhoeven and Elke Lievens performed the experimental work. All authors reviewed and corrected the manuscript.

## Conflict of interest

None declared.

## Supporting information


**Fig. S1.** Map of pMEC45 carrying *gfp* (green arrow) and pMEC45‐derived plasmids carrying *mTagBFP2* (dark blue arrow, in pCMPG11260)*, mCherry* (red arrow, in pCMPG11261)*, mVenus* (yellow arrow, in pCMPG11262) or *ecfp* (cyan arrow, in pCMPG11263) under the control of the *L. lactis* inducible *nisA* promoter (P*nisA,* black arrow). The plasmids contain the *L. lactis* pSH71 replicon (*repA* and *repC* genes) and the chloramphenicol resistance cassette (Cm^R^) depicted by grey arrows (adapted from Geoffroy *et al*., 2000).

**Table S1.** Primers used in this study.
