# Supplementary figures and images for: Expression of fluorescent proteins in Lactobacillus rhamnosus to study host–microbe and microbe–microbe interactions
Source: Microb Biotechnol. 2017 Oct 13;11(2):317–31. doi: 10.1111/1751-7915.12872 (PMC5812243; doi:10.1111/1751-7915.12872)

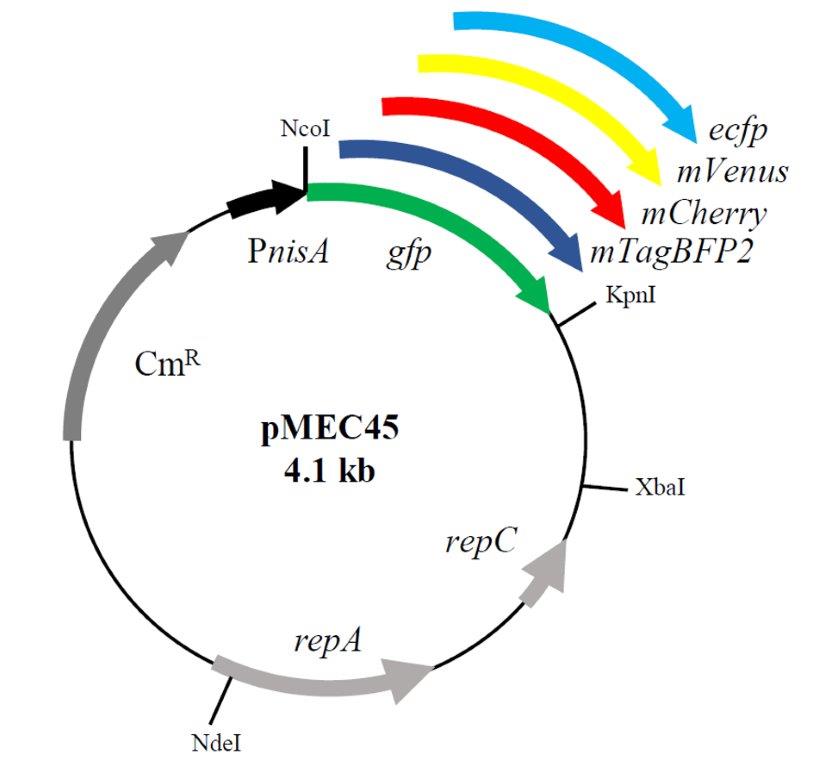

Supplement: Supplementary file 1 — Fig. S1. Map of pMEC45 carrying gfp (green arrow) and pMEC45‐derived plasmids carrying mTagBFP2 (dark blue arrow, in pCMPG11260), mCherry (red arrow, in pCMPG11261), mVenus (yellow arrow, in pCMPG11262) or ecfp (cyan arrow, in pCMPG11263) under the control of the L. lactis inducible nisA promoter (PnisA, black arrow). The plasmids contain the L. lactis pSH71 replicon (repA and repC genes) and the chloramphenicol resistance cassette (CmR) depicted by grey arrows (adapted from Geoffroy et al., 2000). [file MBT2-11-317-s001.jpg]
