# Supplementary material for: Expression of fluorescent proteins in Lactobacillus rhamnosus to study host–microbe and microbe–microbe interactions
Source: Microb Biotechnol. 2017 Oct 13;11(2):317–31. doi: 10.1111/1751-7915.12872 (PMC5812243; doi:10.1111/1751-7915.12872)
Supplement: Supplementary file 2 — Table S1. Primers used in this study. [file MBT2-11-317-s002.docx]

**Table S1. Primers used in this study**

**Primer Sequence Restriction site Remarks**

S&P-01302 ATAAGGAGGCACTCACCATGGTGAGCA / Forward primer *mCherry* for Gibson

AGGGCGAGGAGGA assembly in pMEC45

S&P-01303 CCCACACTACCATCGGTACCTTACTTGT / Reverse primer *mCherry* for Gibson

ACAGCTCGTCCA assembly in pMEC45

S&P-01363 ATCCATGGTGATGGTGAGCAAGGGCGA NcoI Forward primer *mVenus* and *ecfp* for

GG pMEC45

S&P-01364 ATGGTACCTTACTTGTACAGCTCGTC KpnI Reverse primer *mVenus* for pMEC45

S&P-01365 ATGGTACCTCATCGCGACTTGTACAG KpnI Reverse primer *ecfp* for pMEC45

S&P-01366 ATCCATGGTGATGAGCGAGCTGATTA NcoI Forward primer *mTagBFP2* for

AGGAG pMEC45

S&P-01367 ATGGTACCCCGCCAAAACAGCCAAG KpnI Reverse primer *mTagBFP2* for

pMEC45

S&P-00714 TAGAGCTCTTATTTGTAAAGTTCATC / Reverse primer *gfp*

Pro-352 ATTATTGTCGATAACGCGAGCA / Forward primer *nisA* promoter

S&P-01450 CTCTAGCGAGCTGATTAAGGAG / qRT-PCR forward primer *mTagBFP2*

S&P-01451 TTGATTCTCATGGTCTGGGTG / qRT-PCR reverse primer *mTagBFP2*

S&P-01452 AAGCTGAAGGTGACCAAGG / qRT-PCR forward primer *mCherry*

S&P-01453 TTGGAGCCGTACATGAACTG / qRT-PCR reverse primer *mCherry*

S&P-01454 AAAGACCCCAACGAGAAGC / qRT-PCR forward primer *mVenus*

S&P-01455 GTCCATGCCGAGAGTGATC / qRT-PCR reverse primer *mVenus*

S&P-01456 GACAACCACTACCTGAGCAC / qRT-PCR forward primer *ecfp*

S&P-01457 CAGGACCATGTGATCGCG / qRT-PCR reverse primer *ecfp*

S&P-01458 GGTGTTCAATGCTTTTCCCG / qRT-PCR forward primer *gfp*

S&P-01459 CGTCTTGTAGTTCCCGTCATC / qRT-PCR reverse primer *gfp*
